# Efficient second harmonic generation by harnessing bound states in the continuum in semi-nonlinear etchless lithium niobate waveguides

**DOI:** 10.1038/s41377-022-01017-x

**Published:** 2022-11-01

**Authors:** Xueshi Li, Jiantao Ma, Shunfa Liu, Peinian Huang, Bo Chen, Dunzhao Wei, Jin Liu

**Affiliations:** State Key Laboratory of Optoelectronic Materials and Technologies, School of Physics, Sun Yat-Sen University, 510275 Guangzhou, China

**Keywords:** Nonlinear optics, Micro-optics

## Abstract

Integrated photonics provides unprecedented opportunities to pursue advanced nonlinear light sources with low-power consumptions and small footprints in a scalable manner, such as microcombs, chip-scale optical parametric oscillators and integrated quantum light sources. Among a variety of nonlinear optical processes, high-efficiency second harmonic generation (SHG) on-chip is particularly appealing and yet challenging. In this work, we present efficient SHG in highly engineerable semi-nonlinear waveguides consisting of electron-beam resist waveguides and thin-film silicon nitride (SiN)/lithium niobate (LN). By carefully designing octave-separating bound states in the continuum (BICs) for the nonlinear interacting waves in such a hybrid structure, we have simultaneously optimized the losses for both fundamental frequency (FF) and second harmonic (SH) waves and achieved modal phasing matching and maximized the nonlinear modal overlap between the FF and SH waves, which results in an experimental conversion efficiency up to 4.05% W^−1^cm^−2^. Our work provides a versatile and fabrication-friendly platform to explore on-chip nonlinear optical processes with high efficiency in the context of nanophotonics and quantum optics.

## Abstract (web summary)

A semi-nonlinear waveguide in etchless silicon nitride/lithium niobate thin film based on bound states in the continuum achieves high-efficiency second harmonic generation.

## Graphical abstract

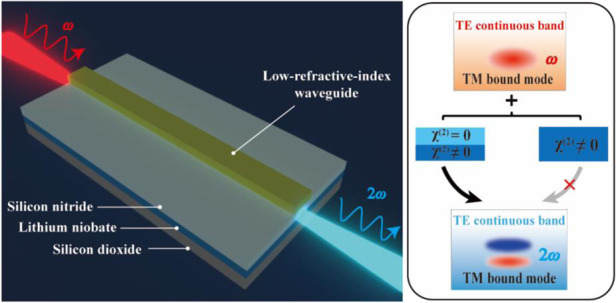

## Introduction

Second-order ($\chi ^{\left( 2 \right)}$) nonlinearities serve as a cornerstone in modern photonics with applications in both classical and quantum regimes such as second-harmonic generation (SHG), sum-frequency generation and difference-frequency generation for frequency conversions and spontaneous parametric down conversion for generating non-classic states of light^1^. In bulk materials, e.g., dihydrogen phosphate (KDP), potassium titanyl phosphate (KTP), lithium niobate (LN), and beta barium borate (BBO), achieving efficient second-order nonlinear optical processes requires high optical powers, phase matching conditions together with long interaction lengths^2^. Empowered by integrated photonics and nanophotonics, chip-scale nonlinear optical devices with low-power consumptions become steadily available due to the greatly enhanced nonlinear light-matter interactions provided by photonic nanostructures^3–5^. In particular, efficient SHG has been obtained in photonic waveguides and micro-resonators made of a variety of materials such as LN, gallium arsenide (GaAa), and aluminum nitride (AlN) with intrinsic $\chi ^{\left( 2 \right)}$ coefficients^6–10^, or silicon (Si) and silicon nitride (SiN) with external-field induced $\chi ^{\left( 2 \right)}$ coefficients^11–13^. Among the material platforms with intrinsic $\chi ^{\left( 2 \right)}$ nonlinearities, the recently developed LN thin film (LNTF) is especially appealing by taking advantages of the excellent material properties of bulk LN and the strong optical confinement and the high-density photonic integration provided by integrated photonics^14–16^. The LNTF devices have exhibited unprecedented performances as electro-optic frequency combs^17,18^, ultra-wide bandwidth modulators^19,20^, low-power nonlinear frequency convertors^21,22^, and high-quality quantum sources^23,24^. For efficient SHG in LNTF waveguides and other $\chi ^{\left( 2 \right)}$ integrated devices, the dispersion engineering enables the same modal refractive index for a fundamental mode serving as the fundamental frequency (FF) wave and one of the higher-order modes serving as the second-harmonic (SH) wave, which is referred to as modal phase matching (MPM) for enhancing the nonlinear optical interactions^25,26^. However, most of the MPM processes suffer from the significant mode profile disparity between the FF and the SH waves, resulting in a small or even vanishing nonlinear modal overlap and consequently limiting the conversion efficiency. Alternatively, quasi-phase matching can be achieved by periodic poling of LN waveguides to maximize the nonlinear modal overlap yet the poling process is not necessarily applicable to other material^27,28^. In addition, the fabrication of periodically poled LNTF waveguides, especially for the *z*-cut one, is still technologically nontrivial^29^.

Recently, bound states in the continuum (BICs) have been proposed and demonstrated as a novel mechanism to achieve on-chip light guiding by patterning low-refractive-index (LRI) structures on a high-refractive-index substrate. In such a device, the light field is guided by the LRI structures but confined in the substrate^30,31^. This new type of BICs is particularly appealing for making integrated photonic devices from the fabrication-challenging materials such as LNTF. The etchless LNTF functional photonic devices were firstly proposed by Zou et al.^30^, and recently demonstrated for high-dimensional communications^32^ and thermal stable SHG^33^.

In this work, we present the realization of high-efficiency SHG in etchless semi-nonlinear SiN/LN photonic waveguides. By very carefully engineering two octave-separating BICs modes supported in such a hybrid device, optimized propagation losses, MPM and large nonlinear modal overlaps for the nonlinear interacting waves are simultaneously achieved, leading to efficient SHG with a normalized conversion efficiency up to 4.05% W^−1^cm^−2^. Our fabrication-friendly platform may immediately facilitate the pursuing of highly-efficient second-order nonlinear processes for on-chip frequency conversions and quantum light generations.

## Results

**Fig. 1:**
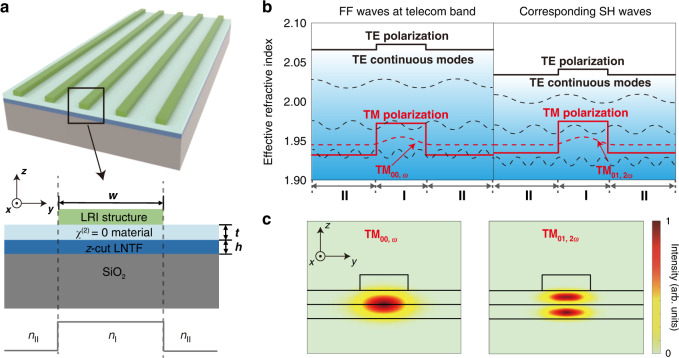
Design of BIC-based etchless semi-nonlinear photonic waveguides. **a** 3D schematic of the BIC-based semi-nonlinear photonic waveguide with the corresponding *y*-*z* cross section. The waveguide consists of a *z*-cut LNTF with a thickness of *h* on a SiO_2_ substrate, a zero-*χ*^(2)^ material with a thickness of *t* and a LRI waveguide with a width of *w* on top. The nonlinear waveguide could be seen as a “three-layers” slab waveguide along *y* axis with equivalent refractive indices of *n*_II,_ *n*_I_ and *n*_II_ from left to right. **b** Effective one-dimensional refractive index along *y*-direction in the corresponding regions of I and II of the waveguide working at the FF and corresponding SH wavelengths. The bound FF TM_00_ and SH TM_01_ modes are engineered to share the same modal refractive index. **c** Mode profiles of the FF TM_00_ and SH TM_01_ bound modes that meet the MPM condition

Figure 1a shows a schematic of the BIC-based etchless semi-nonlinear waveguide consisting of a patterned LRI layer on a zero-$\chi ^{\left( 2 \right)}$ thin film and a LNTF with a silicon oxide (SiO_2_) substrate. The refractive index of the zero-$\chi ^{\left( 2 \right)}$ material is almost the same as the extraordinary refractive index of the LNTF. Their combination forms a semi-nonlinear region with a high refractive index to confine optical fields guided by the LRI waveguide. The light guiding mechanism can be modeled by using an effective one-dimensional refractive index model in which the region with the LRI layer has a higher effective refractive index *n*_I_ than *n*_II_ of the regions on both sides (see details in Supplementary Section 1). As shown in Fig. 1b, the patterned waveguide structure forms a channel with a high effective refractive index to confine light laterally, i.e. the black and red solid lines represent the effective refractive indices for transverse electric (TE) and transverse magnetic (TM) modes, respectively. Such effective refractive index configurations support both discrete modes bounded to the waveguide and extended modes forming the continuum bands. Due to the modal dispersion, modal refractive indices of bound TM modes (red dotted line) are much smaller than that of the fundamental TE mode, resulting in their immersion in the band of continuous high-loss TE modes (black dotted line). In general, the TM modes are lossy due to the coupling to the continuous and extended TE modes. However, by carefully engineering the shape of the waveguide to construct destructive interferences between different coupling channels from the bound TM modes to extended TE modes, the bound TM modes could be decoupled from the continuum of the extended TE modes to form low-loss BICs^30^. The BIC-based waveguide modes ideally encounter a zero propagation loss and could be designed to work at different mode orders for achieving MPM nonlinear interactions. In our proposal, the bound FF TM_00_ and SH TM_01_ modes, whose mode profiles are shown in Fig. 1(c), are carefully engineered to be the BICs with the same modal refractive index for satisfying the MPM condition as shown in Fig. 1b. Their nonlinear interactions in our design rely on the maximal nonlinear coefficient *d*_33_ of the LN, which is significantly larger than *d*_31_ used for other MPM processes. With the assistance of the semi-nonlinear region, the normalized conversion efficiency of SHG, defined by $P_{2\omega }/\left( {P_\omega ^2L^2} \right)$, where $P_\omega$ and $P_{2\omega }$ are the powers of FF and SH waves, respectively, and *L* is the waveguide length, can be expressed as

1 $$\frac{{P_{{{{\mathrm{2}}}}\omega }}}{{P_\omega ^2L^2}} = \frac{{8\pi ^2}}{{\varepsilon _0cn_\omega ^2n_{{{{\mathrm{2}}}}\omega }\lambda _\omega ^2}}\frac{{\xi ^2}}{{A_\omega ^2A_{2\omega }}}$$

here, $n_\omega$ and $n_{2\omega }$ are the modal refractive indices of the FF and SH waves, respectively, $\lambda _\omega$ is the wavelength of the FF wave, $\varepsilon _0$ is the permittivity, and *c* is the speed of light in vacuum. $\xi$ is the nonlinear modal overlap integral, given by

2 $$\xi = d_{{{{\mathrm{33}}}}}\int\!\!\!\int _{{{{\mathrm{LN}}}}}(E_{\omega {{{\mathrm{,z}}}}}^ \ast )^2E_{{{{\mathrm{2}}}}\omega {{{\mathrm{,z}}}}}{\it{dydz}}$$

and $A_\omega$ (or $A_{2\omega }$) is the mode size of the FF TM_00_ (or SH TM_01_) mode, expressed as

3 $$A_\omega = \int\!\!\!\int _{{{{\mathrm{all}}}}}|E_\omega |^2{\it{dydz}}\;\left( {{{{\mathrm{or}}}}\;A_{2\omega }\,{{{\mathrm{ = }}}}\int\!\!\!\int _{{{{\mathrm{all}}}}}|E_{2\omega }|^2{\it{dydz}}} \right)$$

where $E_\omega$ and $E_{2\omega }$ are the electric fields of the FF TM_00_ and SH TM_01_ modes, respectively, $E_{\omega {{{\mathrm{,z}}}}}$ and $E_{2\omega {{{\mathrm{,z}}}}}$ are corresponding *z*-polarization components of $E_\omega$ and $E_{2\omega }$.$\int\!\!\!\int _{{{{\mathrm{all}}}}}$ and $\int\!\!\!\int _{{{{\mathrm{LN}}}}}$ denote integrations over the whole transverse plane and the LN region, respectively. Equation (1) to (3) reveal that efficient SHG requires small mode sizes and a large nonlinear modal overlap integral including a large nonlinear coefficient. Here should be noticed that $\xi$ in above MPM scheme is close to zero if the $\chi ^{\left( 2 \right)}$ material is a homogenous LN because of the odd symmetry of the SH TM_01_ mode^34,35^. In our design, the introduction of a semi-nonlinear region breaks the limit in the nonlinear modal overlap integral in the homogenous nonlinear material, avoiding the counteraction between positive and negative parts of $E_{2\omega }$. For simplicity, we define a factor of $\beta \equiv \xi /\sqrt {A_\omega ^2A_{2\omega }}$to simultaneously optimize the nonlinear modal integral and the mode sizes, so that the normalized conversion efficiency of SHG is proportional to $\beta ^2$.

**Fig. 2:**
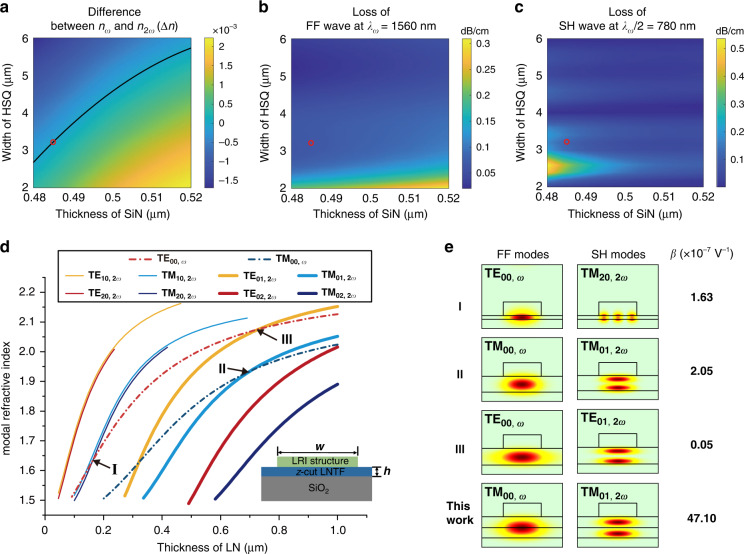
Numerical optimizations of BIC-based MPM processes for nonlinear interacting waves. **a** Difference of modal refractive indices ($\Delta n$) between the FF TM_00_ and SH TM_01_ modes as functions of *w* and *t* at the pump wavelength of 1560 nm. The black line denotes $\Delta n = 0$, indicating the satisfaction of MPM condition. **b**, **c** Corresponding propagation losses dependences on the same parameters in **a** for the FF TM_00_ and SH TM_01_ modes, respectively. The red dots in (**a**–**c**) denotes the chosen parameters in the experiment for nonlinear MPM BICs with small mode sizes and low propagation losses. **d** Dispersions of the FF and SH waves associated to different orders of modes in a pure etchless LNTF waveguide (inset). The intersection points between the FF modes and SH modes refer to all the possible MPM conditions. **e** Comparison of the nonlinear mode overlap between our devices and other MPM conditions in a pure LNTF waveguide

Considering the fabrication process to be compatible with the mature complementary metal-oxide-semiconductor (CMOS) fabrication technology, SiN material (*n* ≈ 2) is chosen to be the zero-$\chi ^{\left( 2 \right)}$ material, while silsesquioxane (HSQ) electron-beam resist (*n* ≈ 1.45) is used as the LRI material to form a waveguide structure. Based on the refractive indices of the chosen materials, we roughly determined thicknesses of the LN and SiN layers that support the MPM condition by calculating modal refractive index based on the effective index theory (see Supplementary Section 1 for details). The thickness of the *z*-cut LNTF is chosen to be *h* = 450 nm meanwhile remaining parameters including the HSQ waveguide width of *w* and the SiN thickness of *t* were globally optimized to engineer losses, mode sizes and dispersions of the FF TM_00_ and SH TM_01_ modes. Aiming at the telecom FF wavelength of 1560 nm, the ranges of *w* and *t* were set from 2 μm to 6 μm and from 0.48 μm to 0.52 μm, respectively, ensuring the co-existences of both octave-separating BIC modes and their MPM condition. The simulated range of *t* also takes into the account of the thickness uncertainty in the SiN thin film deposition process. Figure 2a shows the difference of the modal refractive indices between FF TM_00_ and SH TM_01_ modes as functions of both *w* and *t* for the pump wavelength at 1560 nm. The zero positions in Fig. 2a, where MPM condition is satisfied, are marked by the black line. Clearly, the MPM is more sensitive to the thickness of the SiN than the width of the HSQ waveguide because a higher order mode in *z*-direction instead of *y*-direction is used. The propagation losses of FF and SH waves are shown in Fig. 2b, c, respectively. The SH wave with one-half the wavelength of the FF wave presents a faster oscillation of the propagation loss dependence on *w* (see Supplementary Section 4 for details). For certain *w*, both of the FF TM_00_ and SH TM_01_ modes become nearly lossless BICs. To simultaneously satisfying the MPM condition in Fig. 2a, minimizing the mode area and maintaining low-losses of FF and SH waves in Fig. 2b, c, we chose *t* = 485 nm and *w* = 3.2 μm as the optimized device parameters for SHG. Figure 2d further presents modal refractive indices of the FF and SH waves associated to different orders of modes in a pure etchless LNTF waveguide, in which the intersections between the FF TE_00_ or TM_00_ mode with higher-order SH modes indicate all the possible MPM schemes. However, because of the zero nonlinear coefficient of *d*_23_ = 0 and strictly odd symmetry for TM_10_ and TE_10_ modes, only three MPM processes marked by Roman numerals in Fig. 2d present non-zero nonlinear modal overlaps. We note that the semi-nonlinear hybrid structure can also increase nonlinear modal overlap between FF TE_00_ and SH TE_01_ modes, the smallest coefficient *d*_22_ however limits its conversion efficiency. The mode patterns and values of *β* in these MPM processes are presented in Fig. 2e along with the optimized result of our semi-nonlinear waveguide for a comparison. Among all the possible MPM schemes in the etchless TFLN waveguide, our design features orders of magnitude higher value of *β* owing to its advantages including engineerable $\chi ^{\left( 2 \right)}$ distribution along *z* direction and the employment of the relatively large nonlinear coefficient *d*_33_. According to Eq. (1), the value of $\beta \,{{{\mathrm{ = 47}}}}{{{\mathrm{.10}}}} \times {{{\mathrm{10}}}}^{ - 7}\;V^{ - 1}$ contributes to a theoretical normalized conversion efficiency up to 327% W^-1^cm^-2^ for our device (see Supplementary Section 2 for details). This efficiency could be further increased by guiding the nonlinear interacting BICs on double layer LNTF with internally reversed polarizations^35^ or the periodically poled PPLN without involving any dry etching process^36^. Compared with the recently reported BIC-based nonlinear waveguide composed of LRI materials on a LNTF as case I in Fig. 2(e) shown^33^, our three-layer design presents unique advantages, including designing BIC states for both of FF TM_00_ and SH TM_01_ modes and achieving larger nonlinear modal overlap assisted by the transverse $\chi ^{\left( 2 \right)}$ modulation and the involved largest nonlinear coefficient *d*_33_, for more efficient SHG.

**Fig. 3:**
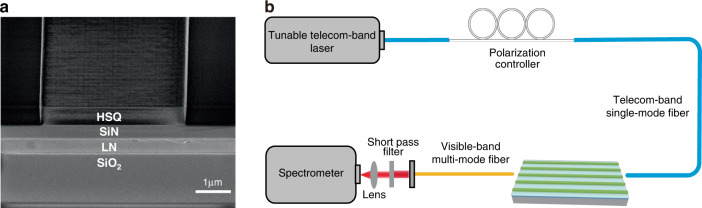
Device fabrication and characterization. **a** SEM image of the cross section of the semi-nonlinear waveguide, showing the LN/SiN layers together with the electron-beam resist waveguide. **b** Experimental setup for the characterization of SHG, which includes an automatic fiber-to-waveguide coupling system, a tunable telecom-band laser and a spectrometer

The devices were fabricated by depositing a 485-nm-thick SiN layer and subsequently spinning a 500-nm-thick HSQ electron-beam resist on top of a commercially available 450-nm-thick *z*-cut LNTF. 2.5 mm-long waveguides with width of 3 μm, 3.2 μm, 3.4 μm, 3.6 μm, and 3.8 μm were patterned in the HSQ layer by electron-beam lithography without involving any dry etching process. The cross section of a representative device was examined by a scanning electron microscope (SEM), as presented in Fig. 3a. Comparing to the widely used nonlinear LN photonic devices, our process is more fabrication-friendly by avoiding the dry etching process for achieving very strong field confinement or ferroelectric domain engineering for compensating phase mismatching. A waveguide-fiber coupling system with a tunable CW telecom-band laser was used to characterize the device and generate the SH wave, as shown in Fig. 3b. A fundamental beam from the laser was coupled into the semi-nonlinear waveguides through a telecom-band single-mode lensed fiber. Its polarization was kept to be consistent with that of the TM mode by adjusting a fiber polarization controller, while its power was directly controlled at the laser source to study power dependences of SHG. At the output port of the device, a multimode lensed fiber was used to collect the FF and SH waves. After filtering out the FF wave with a short pass filter, the SH wave was injected into a spectrometer to measure its spectra and power.

**Fig. 4:**
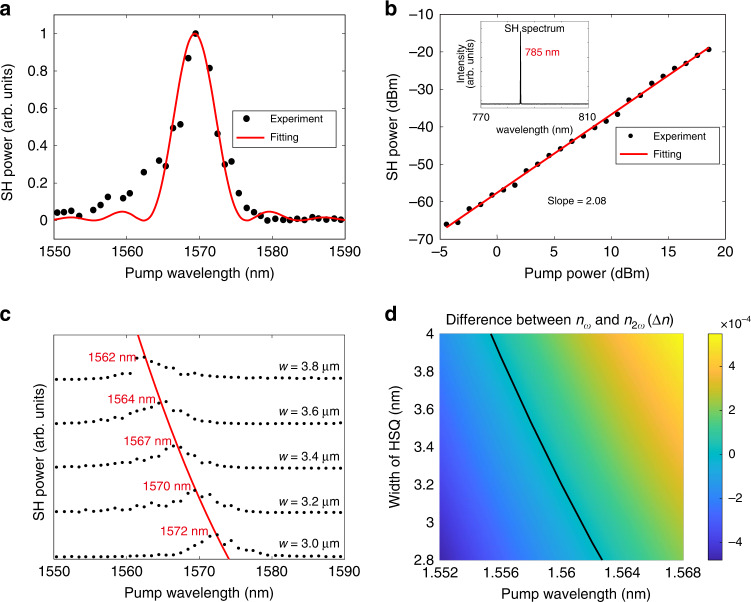
Experimental results of MPM SHG. **a** Nonlinear emission intensity as a function of pumping wavelength with a sinc^2^-function fitting. **b** Power dependence of the generated nonlinear signals at the pumping wavelength of 1570 nm, exhibiting a quadratic relation. Inset: spectrum of the frequency-doubled nonlinear signal. **c** Measured second-harmonic powers as functions of the pump wavelength for the five waveguides width difference widths. **d** Difference of modal refractive indices ($\Delta n$) between the FF TM_00_ and SH TM_01_ modes as a function of the parameters *w* and $\lambda _\omega$, where the black line shows the MPM wavelength changes with the HSQ width

Figure 4a plots the experimental SH intensity as a function of the pumping wavelength for a 3.2 μm-width waveguide. The wavelength dependence of the SH intensity can be well fitted by a sinc^2^-function, confirming that the SHG is enhanced by the MPM nonlinear interactions in the semi-nonlinear waveguide. Fixing the pump laser at the wavelength of 1570 nm at which the highest conversion efficiency is expected, a strong emission at 785 nm was recorded by the spectrometer as the inset presented in Fig. 4b, showing the doubling of the emission frequency. The 10 nm discrepancy between the experimental and simulated MPM wavelengths has little influence on the optimized BIC states owing to its insensitive to wavelengths (see Supplementary Section 5 for details). The power dependence exhibits a quadratic relation in accord with the nature of SHG as shown in Fig. 4b. Figure 4c shows the SHG intensities verse the pumping wavelength for HSQ waveguides with different widths. The peak intensity of the SHG redshifts with the decrease of the waveguide width. Such a trend quantitively follows the simulated MPM condition in Fig. 4d. We further calibrated the normalized conversion efficiency for the waveguide with 3.2-μm-width in which the two MPM BICs experience low propagation losses. Under the pump power of 1 W, the generated SHG reached 1.4 μW, which was enhanced by a factor of ~100 in comparison to the phase-mismatch counterpart. Considering the system losses including end-scattering losses of 5.75 dB for each of the unpolished waveguide facet and end-coupling losses due to the modal mismatch between the semi-nonlinear waveguide and lensed fibers, i.e. 11.45 dB for the FF TM_00_ mode at the input port and 9.65 dB for SH TM_01_ mode at the output port, we obtained an on-chip normalized conversion efficiency of 4.05% W^−1^cm^−2^ (see Supplementary Section 3 for details). Comparing to the theoretical efficiency of 327% W^−1^cm^−2^, the lower efficiency in the experiment mainly attributes to the absorption and scattering losses of the FF and SH waves when propagating in the waveguides. In the experiment, the imperfect attachment between the deposited SiN layer and the LNTF led to the roughness at the interface, resulting in the unavoidable scattering loss during light propagation. Using the cut-back method, the propagation loss of 10 dB cm^−1^ for the FF wave was extracted. The propagation loss for the SH wave is hard to be directly measured. However, according to the discrepancy between the theoretical and experimental normalized conversion efficiencies, the propagation loss of the SH wave was estimated to be ~15 dB cm^-1^. This higher loss at visible regime has been reported due to the undesired defect formed in the SiN layer by the low-temperature chemical vapor deposition^37^. By using more advanced SiN deposition process, e.g., low pressure chemical vapor deposition with high temperature for achieving low material losses, we could further push the conversion efficiency in experiment to the theoretical values. What’s more, bending the waveguide into a microring resonator is potential to improve the normalized conversion efficiency^3^.

## Discussion

In conclusion, we have theoretically proposed and experimentally demonstrated the BIC-based semi-nonlinear photonic waveguides on the etchless SiN/LNTF platform, in which one can flexibly engineer two octave-separating BIC-based waveguide modes, the MPM condition and the nonlinear modal overlap for efficient nonlinear interactions between FF and SH waves. The theoretical normalized conversion efficiency of the BIC-based semi-nonlinear waveguide is as high as 327% W^−1^cm^−2^. Experimentally, we have successfully fabricated such a semi-nonlinear etchless waveguide on base of the hybrid SiN/LNTF platform by using a simplified CMOS process involving only thin film deposition and lithography. The measured normalized conversion efficiency in the fabricated device is 4.05% W^−1^cm^−2^, considerably higher than the MPM SHG in etched *z*-cut or etchless LNTF (see Supplementary Tab. S1 for details). The appreciable deviation of the measured efficiency from the theoretical value is mainly due to the absorption or scattering losses induced by the SiN thin film used in this work. Such a limitation could be overcome by using high-quality materials. Moving forward, reducing the mode size or covering a layer with $- \chi ^{\left( 2 \right)}$, e.g. a *z*-cut LNTF with opposite polarizations, on the LNTF could lead to a higher conversion efficiency comparable to a periodically poled LNTF fabricated by highly-complicated domain engineering. Our method based on BICs in an etchless platform could be easily adopted by other materials for nonlinear devices with expanded functionalities. Along with the simplified fabrication process and tunable MPM wavelength, our work may facilitate the developments of highly-efficient $\chi ^{\left( 2 \right)}$ devices for on-chip frequency conversion, microcomb and quantum light sources^38^. For example, because such a BIC state is wavelength-insensitive and periodically dependent on the width of the LRI material, it is possible to engineer multiple low-loss propagation modes at different wavelengths and mode orders in a single waveguide^31^, which could be used to realize electrical modulator, sum-frequency generation or four-wave mixing. Furtherly constructing complex coupling waveguides, non-Hermitian systems may be realized under the assistance of nonlinear parametric gain to explore new physical phenomena^39,40^.

## Materials and methods

### Numerical simulation

The effective index theory was used as an efficient way to calculate the modal refractive indices of the target propagation modes for modal phase matching in the SHG process as the results shown in Fig. 2d, which was realized by self-writing MATLAB codes. More accurate simulations including the propagation losses as shown in Figs. 2a–c and 4d were carried out via the mode analysis in the COMSOL Multiphysics software. The dispersion relations of the materials were extracted from the databases in the COMSOL software.

### Devices fabrication

The device was fabricated by the CMOS-compatible nanofabrication technique. The 450-nm-thick *z*-cut LNTF binding on a 2 μm-thick SiO_2_ layer was commercially provided by NanoLN Corp. Firstly, precursor gases SiH_4_ and N_2_ in a chemical vapor deposition system (PlasmaPro System100 ICP180-CVD, Oxford Instruments) were used to deposit an amorphous SiN layer with a thickness of ~485 nm on the LNTF. Then, a nearly 500-nm-thick HSQ electron-beam resist was coated on the SiN layer with a spinning speed of 2500 r/min. Finally, electron-beam lithography (VISTEC EBPG5000 ES PLUS, Raith) was used to fabricate HSQ waveguides with five different widths of 3 μm, 3.2 μm, 3.4 m, 3.6 μm, and 3.8 μm, respectively. The ultimate length of the semi-nonlinear photonic waveguide was about 2.5 mm after cleavage.

### Optical measurements

A waveguide-fiber coupling system (WAS 2000, Zhejiang Guangjian Electronic Technology Co., Ltd), combined with a tunable CW telecom-band laser (TSL-550, Santec), an erbium doped fiber amplifier (AEDFA-33-B-FA, Amonics), a power meter (MPM-210, Santec), and a spectrometer (PI SP2758, Princeton Instruments), was used to characterize the waveguides. The laser could be tuned from 1500 nm to 1630 nm with an accuracy of 5 pm in wavelength. It has an output power up to 13 dBm and could be furtherly increased to 33 dBm by the erbium doped fiber amplifier serving as the FF beam. The FF beam with its polarization being controlled by a fiber polarization controller (FPC032, Thorlabs) was coupled into the semi-nonlinear waveguides through a telecom-band single-mode lensed fiber to excite the SH wave. At the output port of the waveguides, a multimode lensed fiber with a diameter of 50 μm in core was used to collect the FF and SH waves. After filtering out the FF wave by a short pass filter, the SH wave was injected into the spectrometer to measure its spectra and power. Absorptive neutral density filters before the spectrometer were optional to attenuate the SH wave for keeping the SH power below the limitation of the spectrometer. Replaced the spectrometer with the power meter, one could measure the total transmission loss of the FF wave.

## Supplementary information

Supplementary Information for Efficient second harmonic generation by harnessing bound states in the continuum in semi-nonlinear etchless lithium niobate waveguides

## Data Availability

The data that supports the results within this paper and other findings of the study are available from the corresponding authors upon reasonable request.
