# Supplementary material for: Efficient second harmonic generation by harnessing bound states in the continuum in semi-nonlinear etchless lithium niobate waveguides
Source: Light Sci Appl. 2022 Nov 1;11:317. doi: 10.1038/s41377-022-01017-x (PMC9622896; doi:10.1038/s41377-022-01017-x)
Supplement: Supplementary file 1 — Supplementary Information for Efficient second harmonic generation by harnessing bound states in the continuum in semi-nonlinear etchless lithium niobate waveguides [file 41377_2022_1017_MOESM1_ESM.docx]

**Supplementary Information for**

**Efficient second harmonic generation by harnessing bound states in the continuum in semi-nonlinear etchless lithium niobate waveguides**

Xueshi Li^1^, Jiantao Ma^1^, Shunfa Liu^1^, Peinian Huang^1^, Bo Chen^1^, Dunzhao Wei^1,*^,and Jin Liu^1,*^

^1^State Key Laboratory of Optoelectronic Materials and Technologies, School of Physics, Sun Yat-Sen University, Guangzhou 510275, China

To whom correspondence should be addressed: weidzh@mail.sysu.edu.cn; liujin23@mail.sysu.edu.cn

**Supplementary Section 1 | Determining the thicknesses of the lithium niobite thin film (LNTF) and the silicon nitride (SiN) layer in the semi-nonlinear region**

To satisfy the modal phase matching (MPM) condition between the fundamental-frequency (FF) TM_00_ and second-harmonic (SH) TM_01_ modes, thicknesses of the *z*-cut LNTF and the SiN layer should be carefully designed to satisfy two requirements: MPM and larger nonlinear modal overlap with smaller mode sizes. We calculated modal refractive indices of the FF TM_00_ and SH TM_01_ modes by solving the wave equation in an equivalent slab waveguide based on the effective index theory. As shown in Fig. S1a, the semi-nonlinear waveguide is divided into core and cladding regions labelled by Ⅰ and Ⅱ, respectively. In these regions, the confinement along *z*-direction is the same as that of a slab waveguide, i.e., Regions I and Ⅱ can be seen as a five-layer slab waveguide with the low-refractive-index (LRI) structure and a four-layer slab waveguide without the LRI structure, respectively. Focusing on *z*-direction mode orders of targeted propagation modes, one can calculate the corresponding effective refractive indices of *n*_Ⅰ_ in Region I and *n*_Ⅱ_ in Region Ⅱ. In the semi-nonlinear waveguide, we should calculate the effective refractive indices for the SH TM_01_ and TE_01_ ­modes as well as the FF TM_00_ and TE_00_ modes as shown in Fig. 1b. Then, a “three-layer” slab waveguide structure along *y* direction is formed for further calculating the modal refractive indices of the FF TM_00_ and SH TM_01_ modes in the MPM process. Figure S1b shows the required thickness *t* of the SiN as a function of the thicknesses *h* of the LNTF for supporting the MPM condition at the pump wavelength of 1560 nm. The results show that *t* is supposed to be around 500 nm when the thickness of LN is set to be *h* = 450 nm for a 3 μm-width LRI structure. The zero-*χ*^(2)^ region occupies nearly half of the high-refractive-index region along *z*-axis, which contributes to a large nonlinear modal overlap. In comparison to the nonlinear BIC-based waveguide composed of LRI materials on the LNTF reported by Fan et al. [Ref. S1], our three-layer design provides more freedoms to simultaneously engineer transverse $\text{χ}^{\left( \text{2} \right)}$ distribution and BIC states for both FF and SH waves, which presents unique advantages including low propagation losses for both of FF TM_00_ and SH TM_01_ modes, larger nonlinear modal overlap with the largest nonlinear coefficient *d*_33_ involved, and cascading $\text{χ}^{\left( \text{2} \right)}$ and $\text{χ}^{\left( \text{3} \right)}$ platform for multifunctional devices.


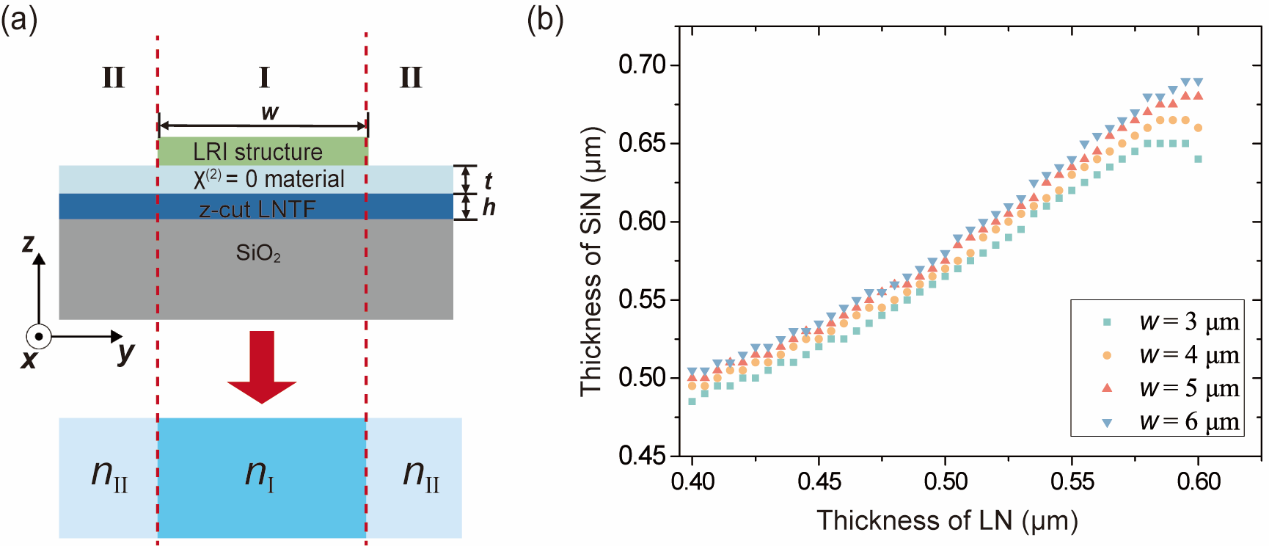


**Figure S1.** Calculating the modal refractive indices for MPM condition based on the effective index theory. (a) Equivalent “three-layer” slab waveguide to our semi-nonlinear waveguide. (b) Thicknesses of the SiN layer for supporting MPM condition as functions of thicknesses of the LNTF for different widths of the LRI waveguides.

**Supplementary Section 2 | Theoretical conversion efficiency of second harmonic generation (SHG) for the semi-nonlinear waveguide based on bound states in the continuum (BICs).**

Ignoring the pump depletion, the normalized conversion efficiency of SHG in a waveguide can be expressed as

$\frac{\text{P}_{\text{2}\text{ω}}}{\text{P}_{\text{ω}}^{\text{2}}\text{L}^{\text{2}}}\text{=}\frac{\text{8}\text{π}^{\text{2}}}{\text{ε}_{\text{0}}\text{c}\text{n}_{\text{ω}}^{\text{2}}\text{n}_{\text{2}\text{ω}}\text{λ}_{\text{ω}}^{\text{2}}}\text{×}\frac{\left| \text{∬}_{\text{all}}\text{χ}^{\left( \text{2} \right)}{(\text{E}_{\text{ω}}^{\text{*}})}^{\text{2}}\text{E}_{\text{2}\text{ω}}\text{dydz} \right|^{\text{2}}}{\left( {\text{∬}_{\text{all}}\left| \text{E}_{\text{ω}} \right|}^{\text{2}}\text{dydz} \right)^{\text{2}}\text{∬}_{\text{all}}\left| \text{E}_{\text{2}\text{ω}} \right|^{\text{2}}\text{dydz}}$ (S1)

In the left-hand side of Eq. (S1), $\text{P}_{\text{ω}}$ and $\text{P}_{\text{2}\text{ω}}$ are the powers of FF and SH waves, respectively, and *L* is the waveguide length. In the right-hand side of Eq. (S1), $\text{n}_{\text{ω}}$ and $\text{n}_{\text{2}\text{ω}}$ are the modal refractive indices of the FF and SH waves, respectively, $\text{λ}_{\text{ω}}$ is the FF wavelength, $\text{ε}_{\text{0}}$ is the permittivity, *c* is the speed of light in vacuum, $\text{E}_{\text{ω}}$ and $\text{E}_{\text{2}\text{ω}}$ are the electric fields of the FF and SH waves, respectively, $\text{χ}^{\left( \text{2} \right)}$ is the second-order nonlinear coefficient, and $\text{∬}_{\text{all}}$ denotes 2D integration over the whole transverse plane. In a MPM process, there is $\text{n}_{\text{ω}}=\text{n}_{\text{2}\text{ω}}$. From Eq. (S1), we can see that the efficiency of SHG depends on the field confinement and the nonlinear modal overlap between FF and SH waves. The nonlinear interaction between the TM modes leads to the effective nonlinear coefficient distribution in the semi-nonlinear region of the waveguide given by

$\text{χ}^{\left( \text{2} \right)}\text{=}\left\{ \begin{matrix} \text{d}_{\text{33}}\text{ In LN}\text{TF}\text{ } \\ \text{0 out of LN}\text{TF}\text{ } \end{matrix} \right.$ (S2)

Therefore, the normalized conversion efficiency can be rewritten as

$\frac{\text{P}_{\text{2}\text{ω}}}{\text{P}_{\text{ω}}^{\text{2}}\text{L}^{\text{2}}}\text{=}\frac{\text{8}\text{π}^{\text{2}}}{\text{ε}_{\text{0}}\text{c}\text{n}_{\text{ω}}^{\text{2}}\text{n}_{\text{2}\text{ω}}\text{λ}_{\text{ω}}^{\text{2}}}\text{×}\frac{\left| \text{d}_{\text{33}}\text{∬}_{\text{LN}}{(\text{E}_{\text{ω}\text{,}\text{z}}^{\text{*}})}^{\text{2}}\text{E}_{\text{2}\text{ω,z}}\text{dydz} \right|^{\text{2}}}{\left( {\text{∬}_{\text{all}}\left| \text{E}_{\text{ω}} \right|}^{\text{2}}\text{dydz} \right)^{\text{2}}\text{∬}_{\text{all}}\left| \text{E}_{\text{2}\text{ω}} \right|^{\text{2}}\text{dydz}}$ (S3)

$\text{E}_{\text{ω,z}}$ and $\text{E}_{\text{2}\text{ω,z}}$ refer to the *z*-polarization component of the FF and SH waves. For the optimized waveguide with a 3.2 μm-width LRI structure, the modal refractive indices of the TM_00_ mode at 1560 nm and TM_01_ mode at 780 nm are the same to support MPM. Their simulated propagation losses are $\text{η}_{\text{ω}}\text{ = 0.066 dB/cm}$ and $\text{η}_{\text{2}\text{ω}}\text{ }\text{= 0.117}\text{ }\text{dB/cm}$, respectively. If$\text{ }\text{d}_{\text{33}}\text{ }\text{= 27.2}\text{ }\text{pm}\text{∙}\text{V}^{\text{-1}}$, The theoretical normalized conversion efficiency of the SHG reaches $\text{327\%}{\text{ }\text{W}}^{\text{-1}}\text{cm}^{\text{-2}}$ based on Eq. (S3).

**Supplementary Section 3 |** **Calculating the experimental normalized conversion efficiency of SHG**

Figure S2a shows the schematic diagram of the experimental setup for SHG characterization. The output SH wave was recorded by a spectrometer. In order to calculate the actual output SH power, we used a reference laser beam at the wavelength of $\text{λ}_{\text{ref}}\text{ }\text{=}\text{ }\text{785 nm}$ with nearly the same bandwidth as that of the SH wave to calibrate the power detection of the spectrometer. We sent the attenuated beam with a power of $\text{P}_{\text{ref}}\text{ = }\text{4.86}\text{ }\text{pW}$ into the spectrometer. The measured photon number is about $\text{N}_{\text{r}\text{ef}}\text{ }\text{=}\text{ }\frac{\int f\left( \lambda\right)d\lambda}{\delta\lambda\Delta t}\text{=}\text{ }\text{5500}\text{0 }\text{s}^{-1}$, where $f\text{(}\text{λ}\text{)}$ is the recorded spectrum, $\delta\lambda$ is the wavelength space, and $\Delta t$ is the acquisition time. According to the relationship between detected power $\text{P}_{\text{D}}$ and the collected photon number expressed as

$\text{P}_{\text{D}}\text{ }\text{=}\text{N}_{\text{ref}}\text{ }\text{h}\frac{\text{c}}{\text{λ}_{\text{ref}}}$ (S4)

where *h* is the Planck constant and *c* is the speed of light. The detected power was estimated to be $\text{P}_{\text{D}}\text{ ≈ 0.0}\text{14}\text{ }\text{pW}$. Therefore, we extracted the transmission coefficient from the collected multimode fiber to the spectrometer, given by,

$\text{η=}\frac{\text{P}_{\text{D}}}{\text{P}_{\text{ref}}}\text{ }\text{=}\text{ }\text{0.288\%}$ (S5)

To further calculate a normalized conversion efficiency of the SHG, one should know end-coupling losses attributed to modal mismatch between the nonlinear waveguide and the lensed fiber, end-scattering losses originated from the unpolished facets and propagation losses of the FF and SH waves, respectively, as shown in Fig. S2b. Firstly, we carried out numerical simulations based on the parameters of the single-mode and multi-mode lensed fibers as well as the 3.2 μm-width waveguide to calculate the end-coupling losses, obtaining the fiber-to-waveguide and waveguide-to-fiber coupling losses of the FF wave as $\text{α}_{\text{ω}\text{,in}}=5.7 dB$ and $\text{α}_{\text{ω}\text{,out}}=2.3 \mathrm{dB}$, respectively, as well as the waveguide-to-fiber coupling loss for the SH wave as $\text{α}_{\text{2}\text{ω}\text{,out}}=3.9 \mathrm{dB}$, presented in Figs. S3a to S3c. Secondly, we used the cut-back method to extract the propagation loss $\text{η}_{\text{ω}}$ of the FF wave from two 3.2 μm-width waveguides with lengths of 1.5 mm and 11 mm. Their transmission losses at the wavelength of 1570 nm measured by a telecom-band power meter were about 19 dB and 28.5 dB, respectively, leading to $\text{η}_{\text{ω}}\approx10 dB\cdot\mathrm{cm}^{-1}$. Thirdly, we used the telecom-band power meter to measure the normalized transmission spectrum of the FF wave for the 3.2 μm-width waveguide performing the SHG as shown in Fig. S2b, which has an average transmission loss of 22 dB. Therefore, the end-scattering loss for the FF wave was calculated to be $\text{α}_{\text{ω}\text{, }\text{sca}}=\frac{22 dB-\text{α}_{\text{ω}\text{,in}}- \text{α}_{\text{ω}\text{,out}}-\text{η}_{\text{ω}}\times0.25 \mathrm{cm}}{2}= 5.75 \mathrm{dB}$, resulting in the total loss $\alpha_{\omega,\mathrm{sca}} + \alpha_{\omega,\mathrm{in}}= 11.45\mathrm{dB}$at the input port. Such high end-scattering losses are revealed by a top-view infrared imaging system shown in Figs. S3e and S3f. It is hard to measure the propagation loss $\text{η}_{\text{2}\text{ω}}$ of the SH wave for calculating its end-scattering loss $\text{α}_{\text{2}\text{ω}\text{, }\text{sca}}$. Here, we assumed $\text{α}_{\text{2}\text{ω}\text{, }\text{sca}}=\text{α}_{\text{ω}\text{, }\text{sca}}$, so that the loss of SH power at the output port was $\alpha_{2\omega,\mathrm{sca}} + \alpha_{2\omega, \mathrm{out}}= 9.65\mathrm{dB}$.

When the pump power reached 1W, the output SH power from the waveguide attenuated by a factor of 10^5^ through absorptive neutral density filters and recorded by the spectrometer. The counting photons corresponded to a detected power of 0.0405 pW, indicating an actual output power of 1.4 μW. Further considering the end-coupling and end-scattering losses of the FF wave at the input port and the SH wave at the output port, the on-chip normalized conversion efficiency was calculated to be 4.05% W^-1^ cm^-2^. Comparing to the theoretical efficiency of 327% W^-1^cm^-2^, the lower efficiency in the experiment mainly attributes to the absorption and scattering losses of the FF and SH waves when propagating in the waveguide. If the experimental $\text{η}_{\text{ω}}\approx10 dB\cdot\mathrm{cm}^{-1}$ is considered, the theoretical normalized conversion efficiency should be revised as 26.8%. Further considering the propagation loss of the SH wave, the theoretical value would become the same as the experimental one if $\text{η}_{\text{2}\text{ω}}\approx15 dB\cdot\mathrm{cm}^{-1}$.

As comparison, Tab. S1 provides the performances of MPM SHG in etch or etchless LN waveguide reported by other literatures so far. The given propagation losses of SH waves in Tab. S1 are mainly provided by estimation, because it is difficult to measure a propagation loss for a high-order mode. With further optimizations on the waveguide loss, e.g., by using low-pressure chemical vapor deposition SiN, we could push the conversion efficiency in the experiment to the theoretic values. Such a number could be further improved by using double layer LNTF with internally reversed polarizations or periodically poled LNTF without involving any dry etching process.


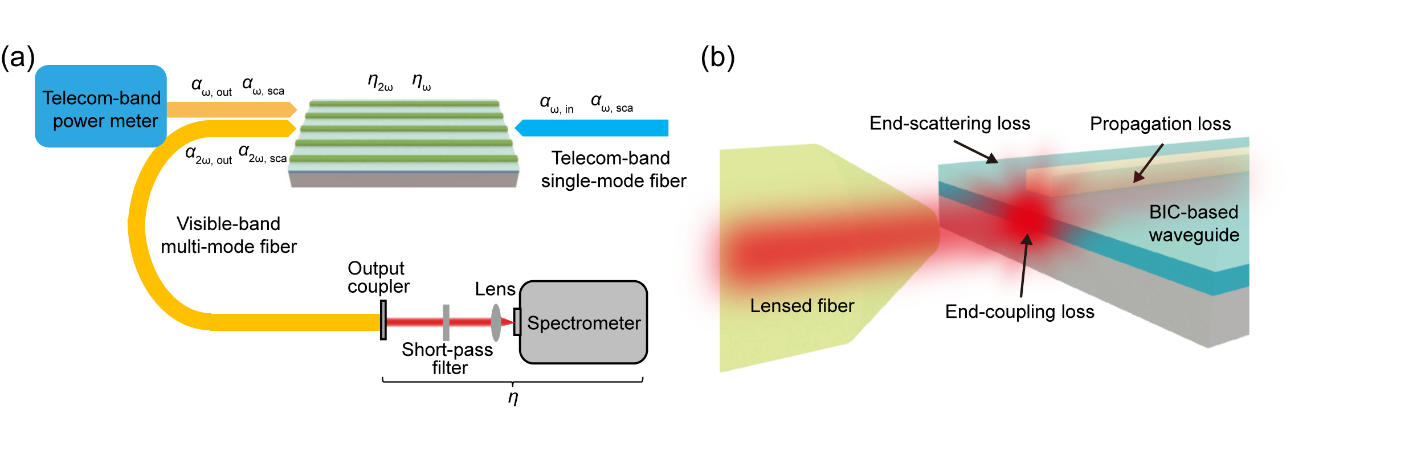


**Figure S2.** (a) Schematic diagram of experimental setup for measuring the on-chip normalized conversion efficiency. The transmitted FF wave was recorded by a telecom-band power meter while the generated SH wave was collected by a spectrometer. (b) Schematic system losses including end-scattering losses from the unpolished facets, end-coupling losses from the modal mismatch between the lensed fiber and the BIC-based waveguide, and propagation losses mainly from the SiN layer.


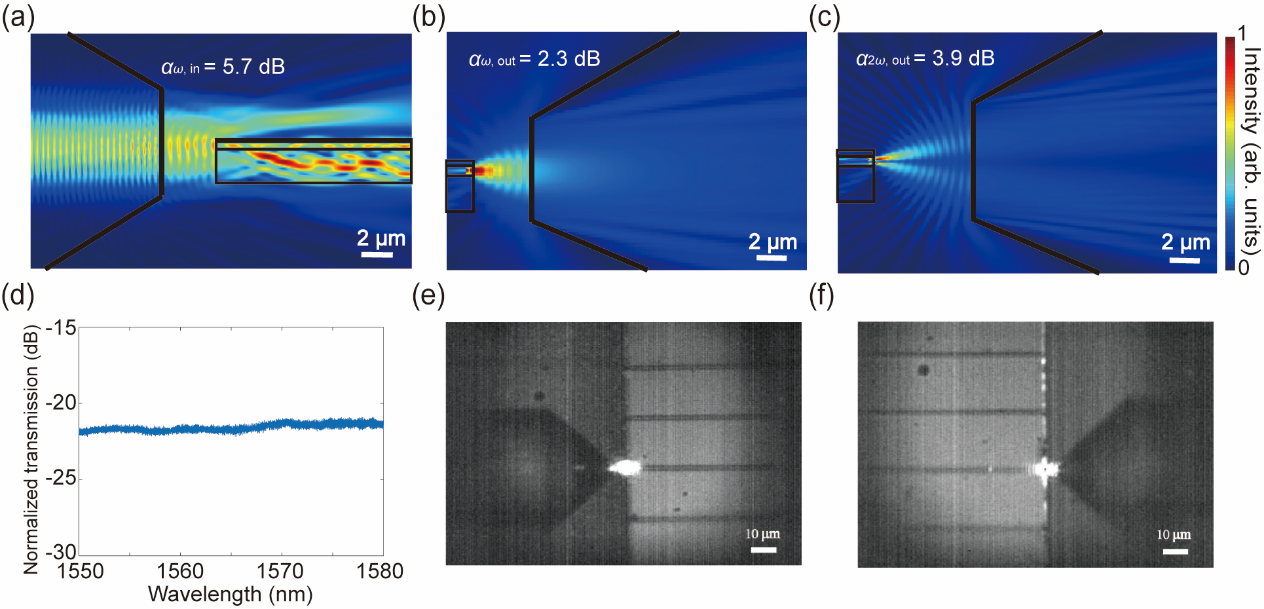


**Figure S3.** (a) to (c) Numerical simulations of end-coupling processes including fiber-to-waveguide coupling for the FF TM_00_ mode ($\text{α}_{\text{ω}\text{,in}}=5.7 dB$) (a), waveguide-to-fiber coupling for the FF TM_00_ mode ($\text{α}_{\text{ω}\text{,out}}=2.3 \mathrm{dB}$) (b), and waveguide-to-fiber coupling for the SH TM_01_ mode ($\text{α}_{\text{2}\text{ω}\text{,out}}=3.9 \mathrm{dB}$) (c). (d) The measured normalized transmission spectrum of the FF waves at the telecom band, indicating a total transmission loss of 22 dB. (e) and (f) Top views of end-coupling processes for the FF wave including fiber-to-waveguide coupling (e) and waveguide-to-fiber coupling (f), revealing strong end-scattering light with a loss of $\text{α}_{\text{ω}\text{, }\text{sca}}= 5.75 \mathrm{dB}$ for each facet.

**Table S1.** Performances of MPM SHG in LN waveguides

| References | LN wafer type | FF to SH mode conversion | Theoretical conversion efficiency  (W^-1^cm^-2^) | Experimental conversion efficiency  (W^-1^cm^-2^) | Waveguide loss at FF wave  (dB·cm^-1^) | Waveguide loss at SH wave  (dB·cm^-1^) | Device length |
| --- | --- | --- | --- | --- | --- | --- | --- |
| Optics express 25(6), 6963(2017) | x-cut (etched) | TE_1,ω_→TE_3, 2ω_ | 60% | 41% | 3.0 | ~ | 1 mm |
| Optica 5(8), 1006(2018) | z-cut (etched) | TE_00,ω_→TM_02, 2ω_ | 22.2% | 7.3% | 0.54 | 2.16  (estimated) | 8 mm |
| Laser & Photonics Reviews 13(3), 1800288(2019) | x-cut (etched) | TE_00,ω_→TE_01, 2ω_ | 2900% | 650% | 3.2 | 12.8  (estimated) | 2.35 mm |
| Laser & Photonics Reviews 15(12), 2100409(2021) | x-cut (etched) | TE_00,ω_→TE_01, 2ω_ | 9825% | 5540% | 3.8 | ~ | 1.2 mm |
| Laser & Photonics Reviews 16(3), 2100429(2022) | z-cut (etchless) | TE_00,ω_→TM_20, 2ω_ | 0.56% | 0.175% | 2 | ~ | 5 mm |
| This work | z-cut (etchless) | TM_00,ω_→TM_01, 2ω_ | 327% | 4.05% | 10 | 15  (estimated) | 2.5 mm |

**Supplementary Section 4 | Decoupling principle between the bound TM modes and continuum TE modes**

As long as the waveguide thickness is smaller than the wavelength, by continuity the propagation modes in the waveguide should be mostly TE-like or TM-like, causing both the TE and TM modes to contain *E_y_* and *E*_z_ components. The difference is that *E*_z_ field dominated in the bound TM mode and *E_y_* field dominated the continuous TE modes, respectively. Therefore, the bound TM mode can interact with the continuous TE modes with propagation loss. The coupling losses mainly happen at two edges of the waveguide, where scattering leads the bounded TM mode to dissipate into each TE mode through four channels, left-going channels 1 and 3 and right-going channels 2 and 4, as shown in Fig. S4. The left-going channels (or right-going channels) will destructively interfere with each other by controlling their phase differences via the waveguide width for certain wavelengths.


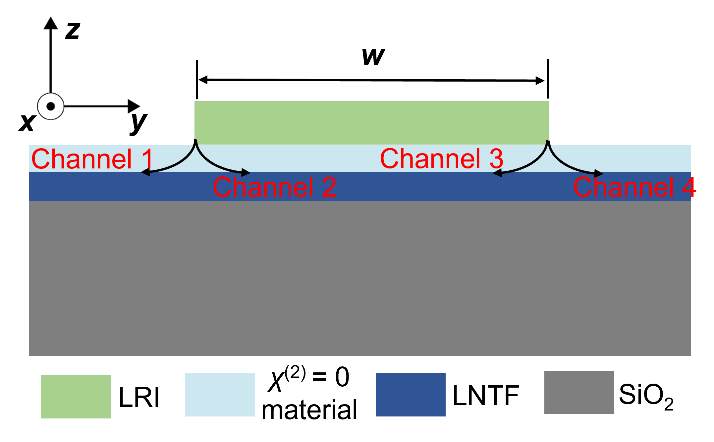


**Figure S4.** Dissipation channels of the bound TM mode to each TE mode

Since the *E*_y_ components of the TM mode at the two edges of the waveguide have a $\pi$-phase difference, the destructive interference between the dissipated Channels 1 and 3 (or Channels 2 and 4) follows a relation $k_{y}w_{\mathrm{BIC}}=2m\pi$ as shown in Fig. S4 [Ref. S2]. Here, *m* is an integer and $k_{y}$ is the transverse wave vector for the FF wave, given by

$k_{y}=\sqrt{n^{2}-{n_{\omega}}^{2}}\frac{2\pi}{\lambda_{\omega}}$ (S6)

where$n$and $n_{\omega}$ are extraordinary refractive index of the LN and modal refractive index at the frequency of *ω*. So that the width of HSQ satisfying BIC states can be expressed as

$w_{\mathrm{BIC}}={m\lambda_{\omega}}/{\sqrt{n^{2}-{n_{\omega}}^{2}}}$ (S7)

Equation (S7) indicates that $w_{\mathrm{BIC}}$ for the BIC points appears periodically. Besides, $w_{\mathrm{BIC}}$ for the SH wave, i.e. $\lambda_{2\omega}$, has nearly one-half period of that for the FF wave as shown in Figs. 2b and 2c, if material dispersion of *n* and modal dispersion of $n_{\omega}$ are ignored. Here should be noticed that $n_{\omega}$ changes with *w*.

**Supplementary Section 5 | Wavelength response of the BIC-based waveguide**

Figure S5 shows the simulated propagation losses dependences on wavelengths of FF and SH waves respectively. The results show that the BIC states are very insensitive to wavelength, which make the devices robust against fabrication deviation or environmental temperature fluctuation.


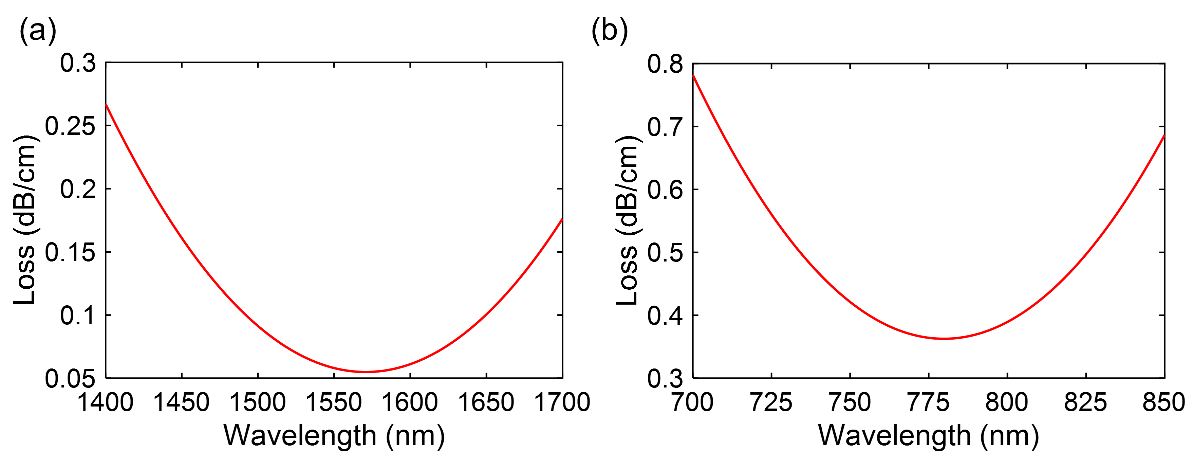


**Figure S5.** Propagation losses dependences on wavelengths for the FF TM_00_ mode (a) and SH TM_01_ mode (b), respectively, with the device parameters of *t* = 485 nm and *w* = 3.2 μm.

**References**

S1 Ye, F., Yu, Y., Xi, X. & Sun, X. Second‐Harmonic Generation in Etchless Lithium Niobate Nanophotonic Waveguides with Bound States in the Continuum. *Laser & Photonics Reviews* **16**, 2100429 (2022).

S2 Zou, C.-L. *et al.* Guiding light through optical bound states in the continuum for ultrahigh-Qmicroresonators. *Laser & Photonics Reviews* **9**, 114-119 (2015).
